# Supplementary figures and images for: Interleukin 35 induced Th2 and Tregs bias under normal conditions in mice
Source: PeerJ. 2018 Sep 21;6:e5638. doi: 10.7717/peerj.5638 (PMC6152461; doi:10.7717/peerj.5638)

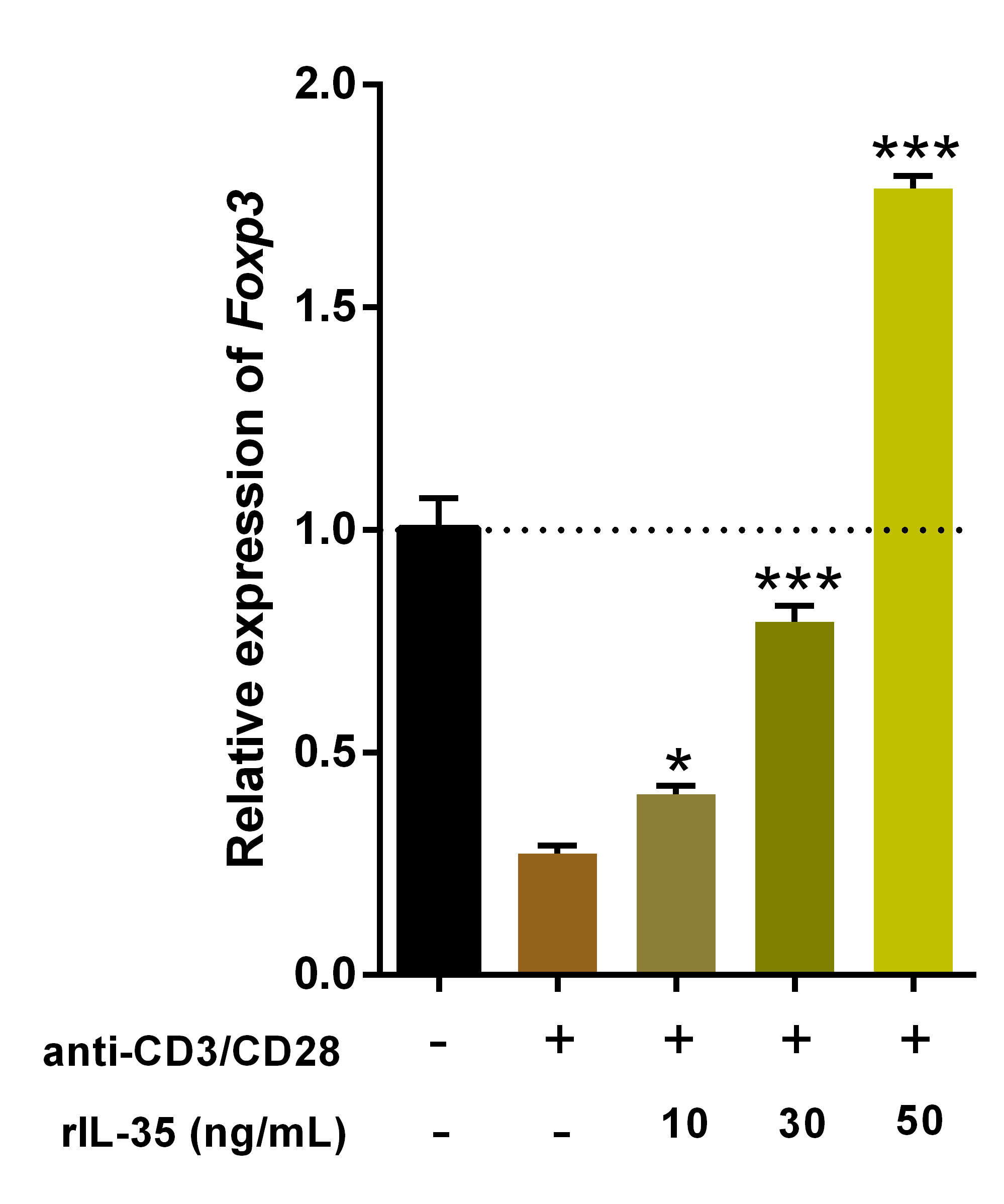

Supplement: Supplemental Information 1 — The expression of Il2, Il10, and Foxp3 in splenic T cells following rIL-35 addition for 24 h in vitro. [file peerj-06-5638-s001.zip › Foxp3 in vitro.png]

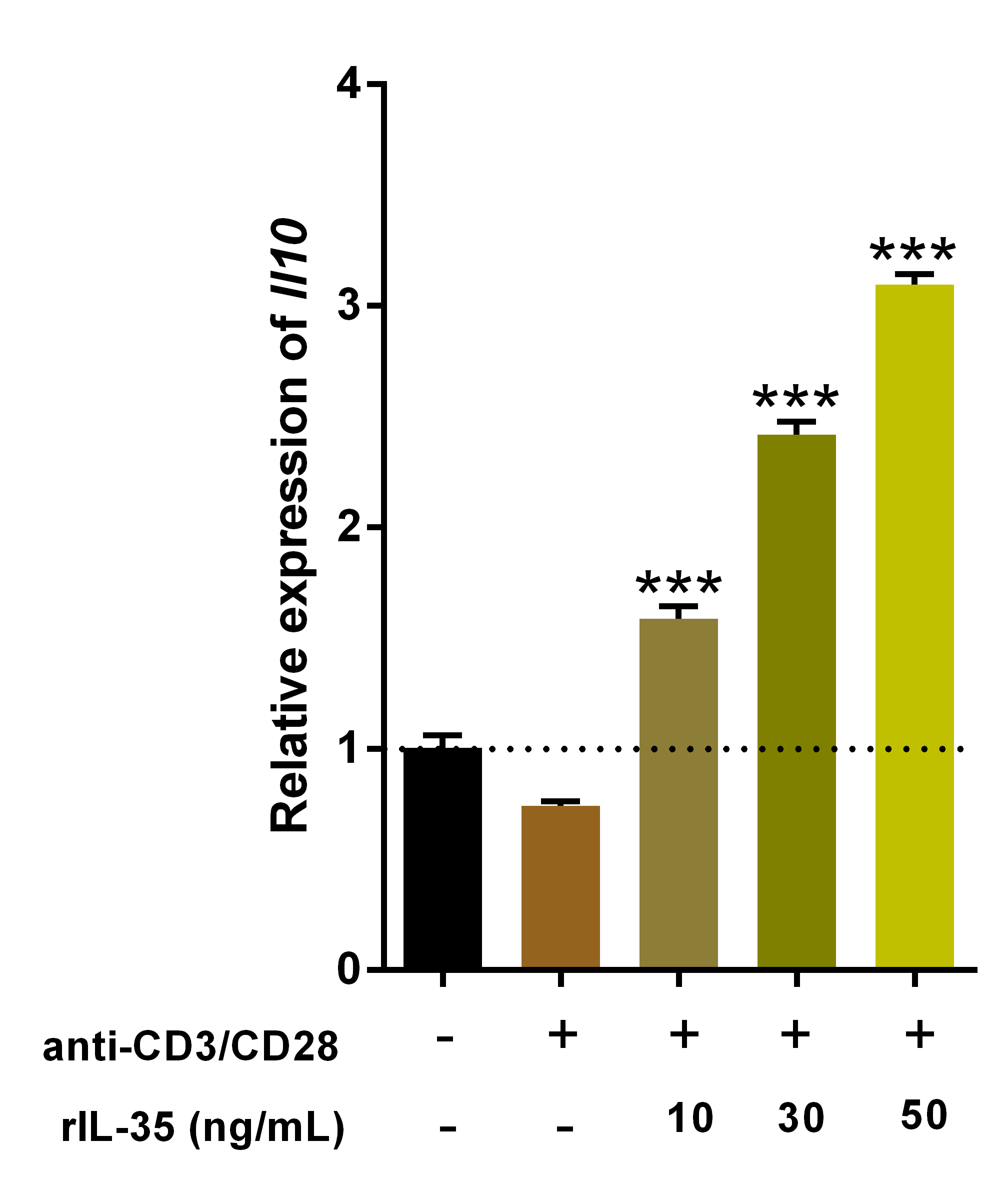

Supplement: Supplemental Information 1 — The expression of Il2, Il10, and Foxp3 in splenic T cells following rIL-35 addition for 24 h in vitro. [file peerj-06-5638-s001.zip › Il10 in vitro.png]

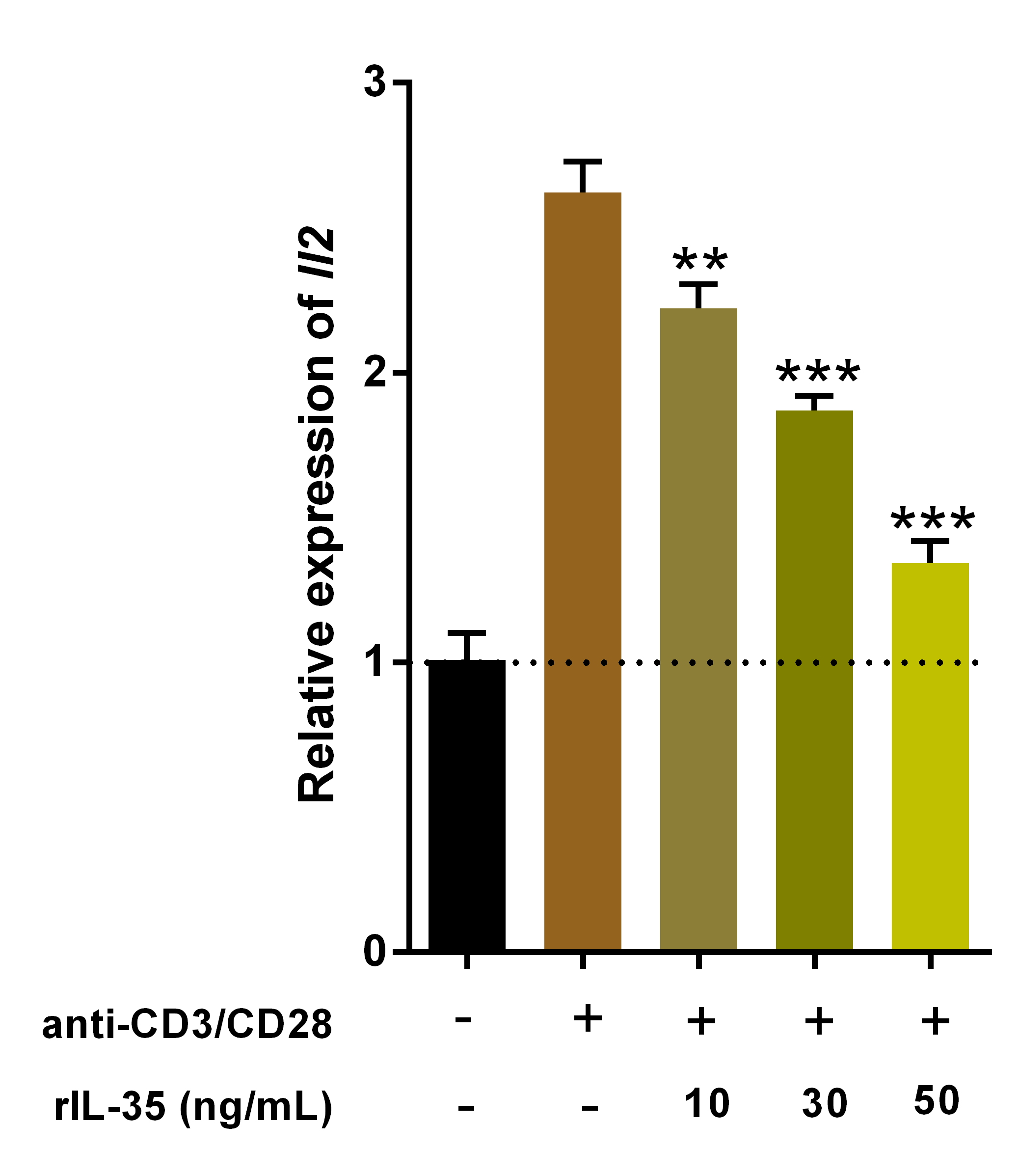

Supplement: Supplemental Information 1 — The expression of Il2, Il10, and Foxp3 in splenic T cells following rIL-35 addition for 24 h in vitro. [file peerj-06-5638-s001.zip › Il2 in vitro.png]

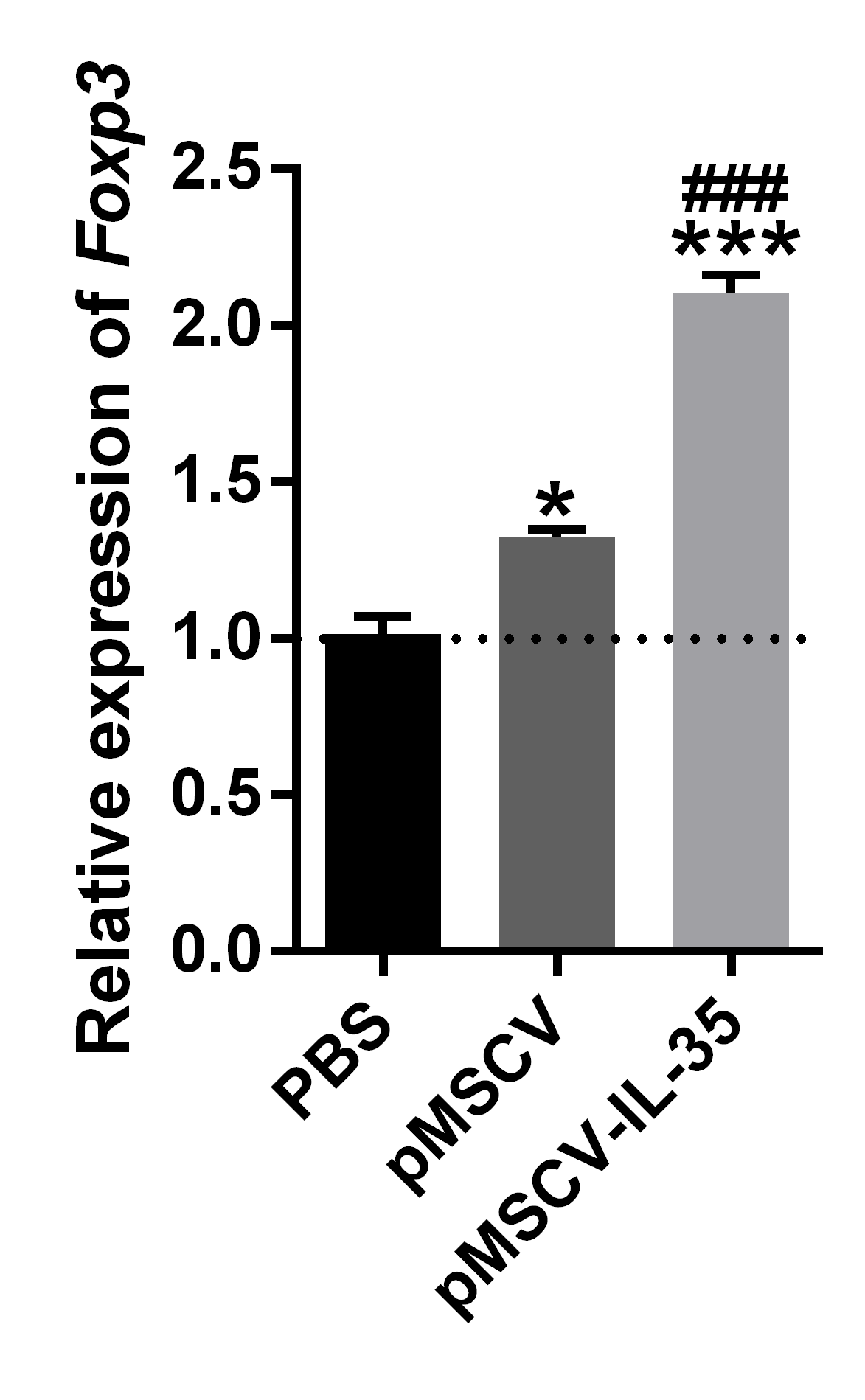

Supplement: Supplemental Information 2 — The mRNA level of Il2, Ifng, Il10, Gzmb, Prf1, and Foxp3 in vivo. [file peerj-06-5638-s002.zip › Foxp3 in vivo.png]

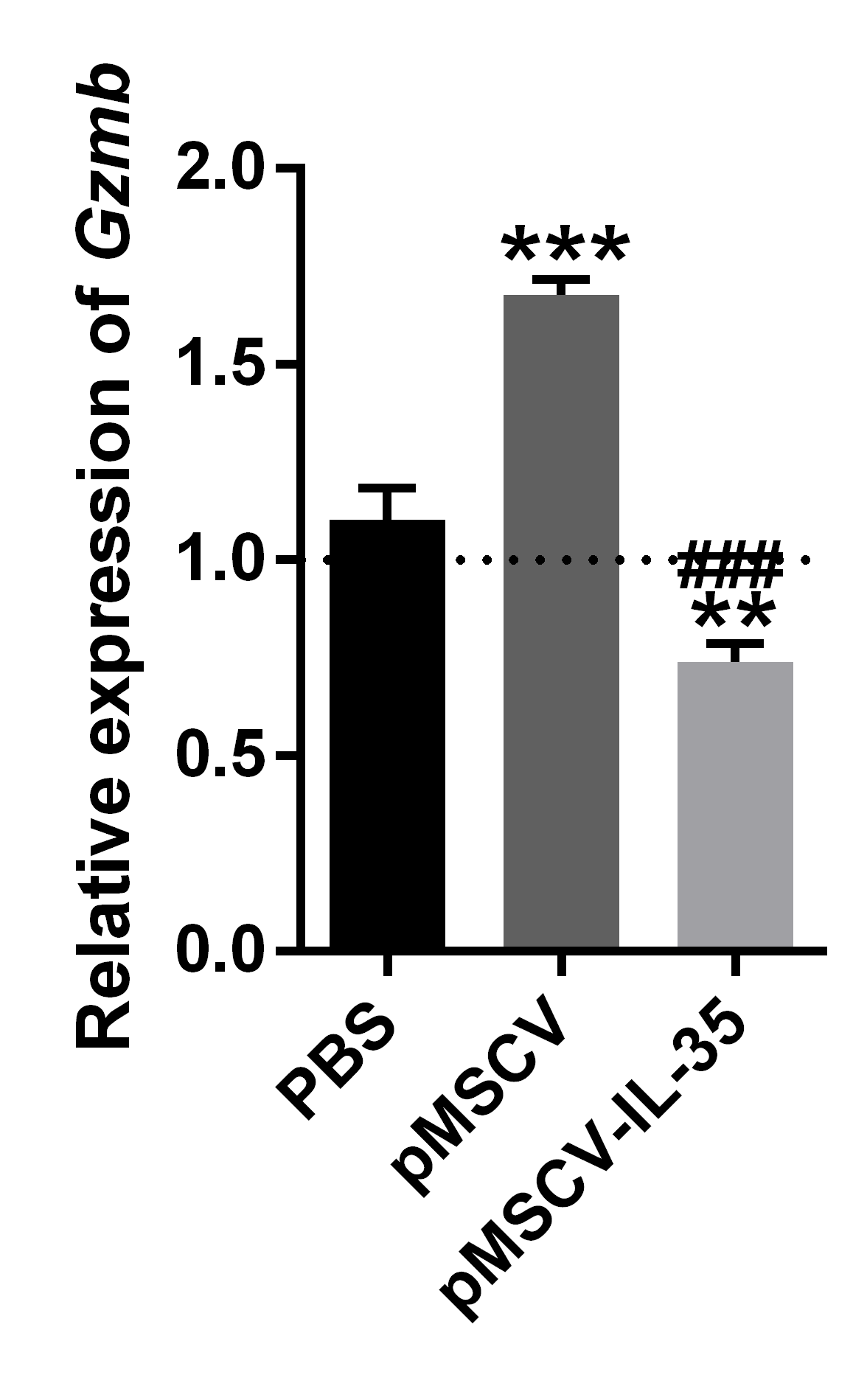

Supplement: Supplemental Information 2 — The mRNA level of Il2, Ifng, Il10, Gzmb, Prf1, and Foxp3 in vivo. [file peerj-06-5638-s002.zip › gzmb in vivo.png]

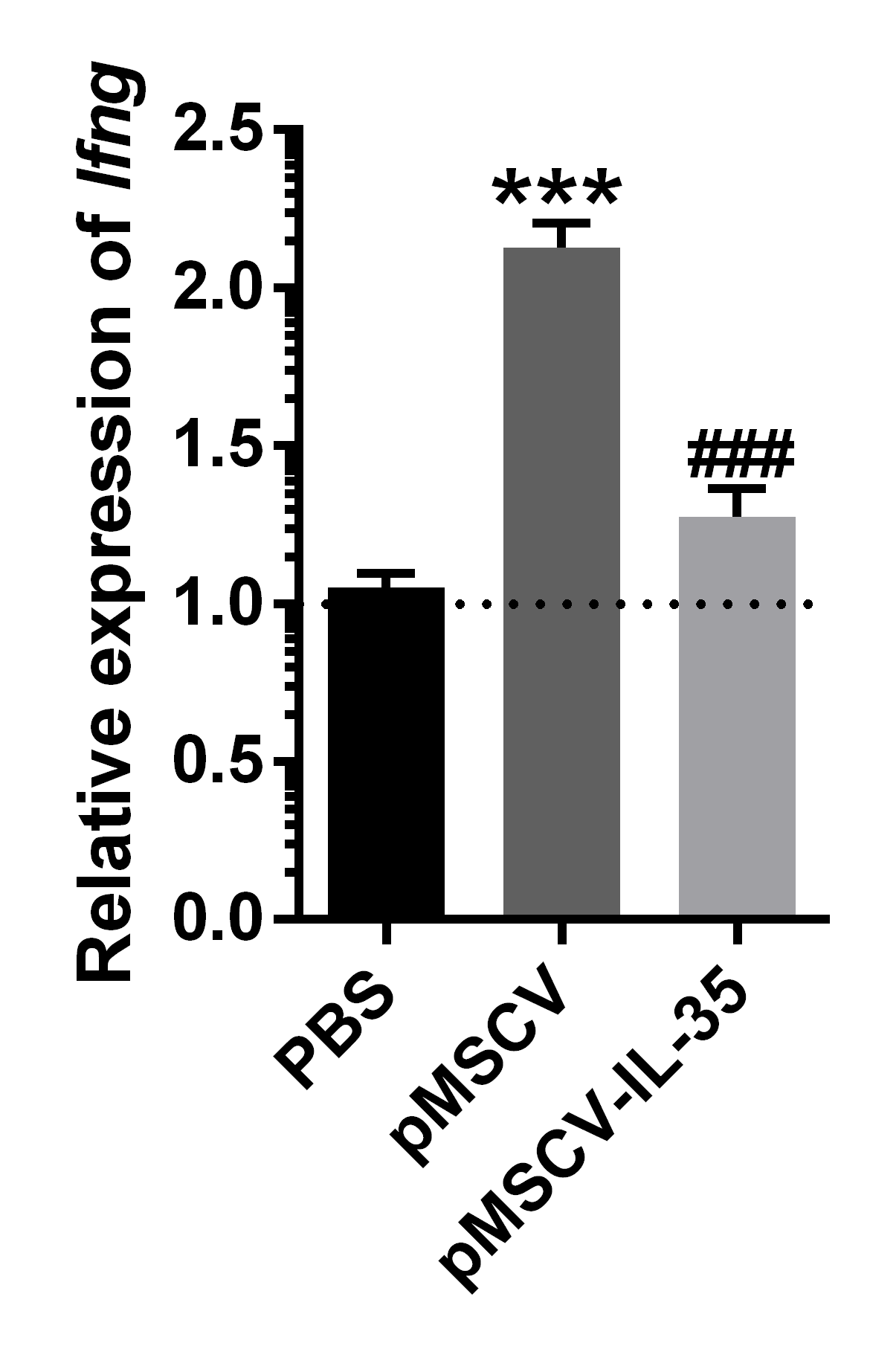

Supplement: Supplemental Information 2 — The mRNA level of Il2, Ifng, Il10, Gzmb, Prf1, and Foxp3 in vivo. [file peerj-06-5638-s002.zip › IFN in vivo.png]

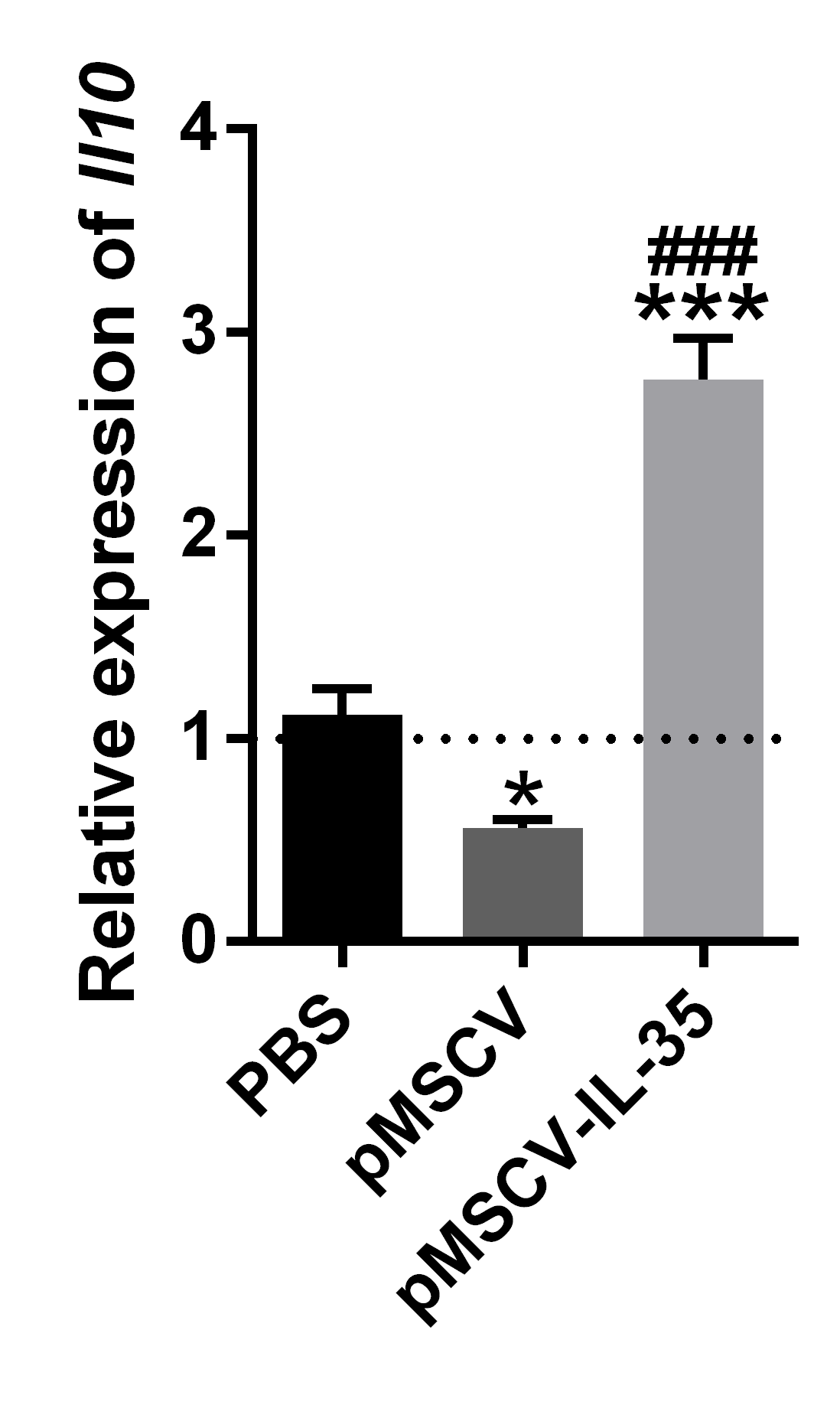

Supplement: Supplemental Information 2 — The mRNA level of Il2, Ifng, Il10, Gzmb, Prf1, and Foxp3 in vivo. [file peerj-06-5638-s002.zip › IL10 in vivo.png]

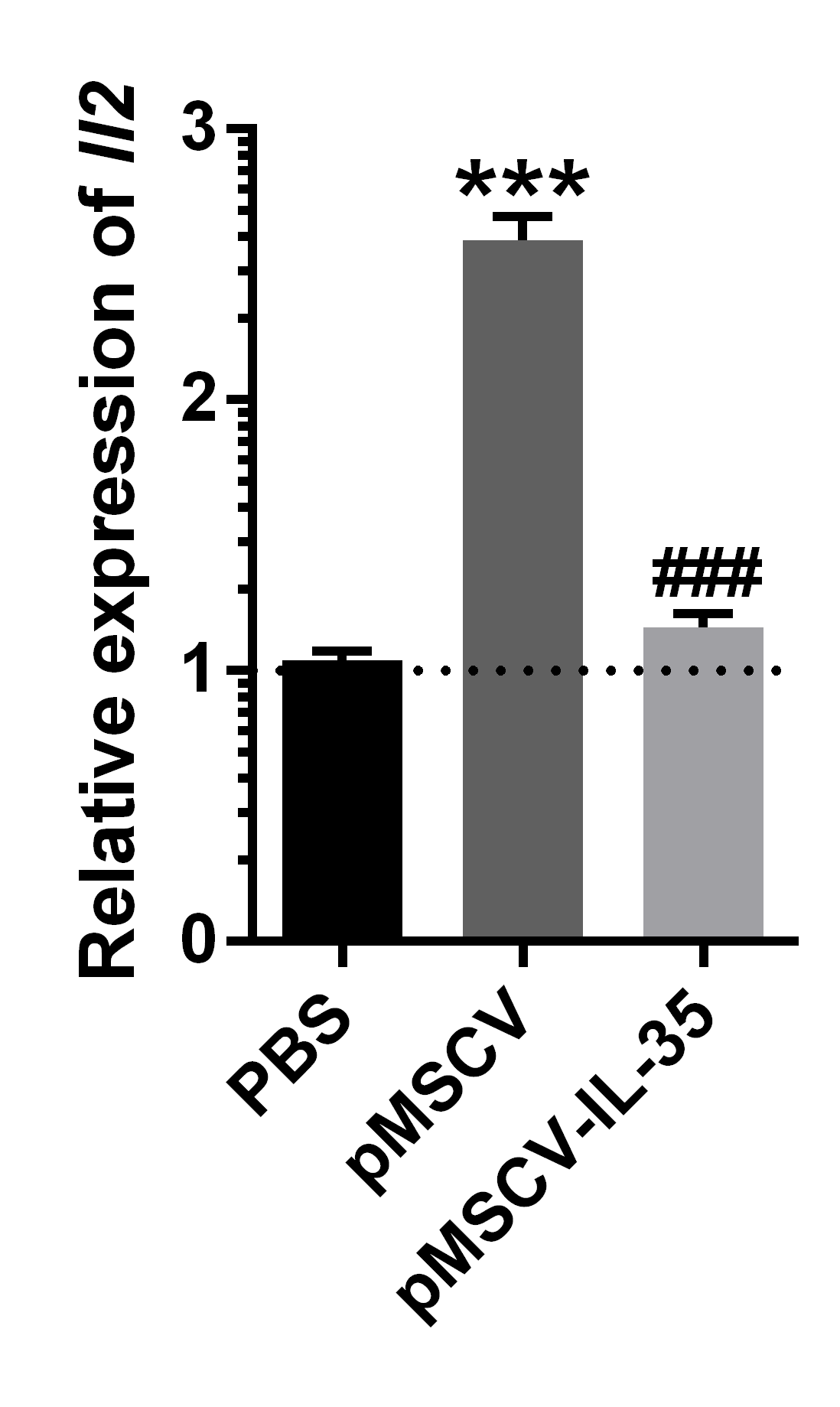

Supplement: Supplemental Information 2 — The mRNA level of Il2, Ifng, Il10, Gzmb, Prf1, and Foxp3 in vivo. [file peerj-06-5638-s002.zip › IL2 in vivo.png]

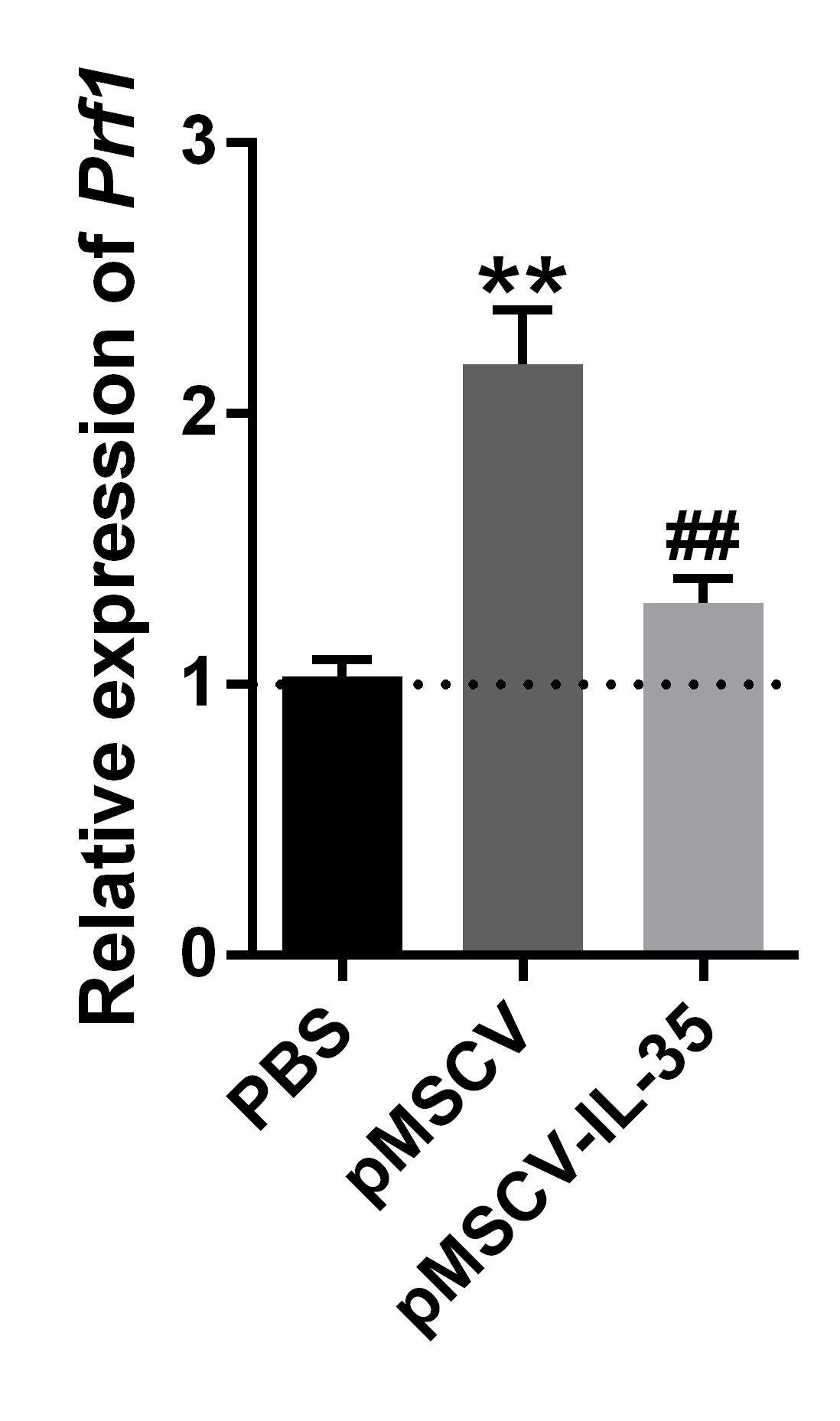

Supplement: Supplemental Information 2 — The mRNA level of Il2, Ifng, Il10, Gzmb, Prf1, and Foxp3 in vivo. [file peerj-06-5638-s002.zip › prf1 in vivo.png]
